# miR-3648 promotes lung adenocarcinoma-genesis by inhibiting SOCS2 (suppressor of cytokine signaling 2)

**DOI:** 10.1080/21655979.2021.2017577

**Published:** 2022-01-17

**Authors:** Yanhong Tu, Fan Mei

**Affiliations:** Department of Geriatrics, The Sixth Hospital of Wuhan, Affiliated Hospital of Jianghan University, Wuhan, Hubei, China

**Keywords:** Lung adenocarcinoma, miR-3648, SOCS2, proliferation, migration, invasion

## Abstract

Lung adenocarcinoma (LUAD) is the most common histologic subtype of lung cancer and is associated with high morbidity and mortality. We aimed to study the effects of microRNA-3648 (miR-3648) on LUAD by inhibiting its downstream target suppressor of cytokine signaling 2 (SOCS2) mRNA. miR-3648 expression was measured by real-time quantitative PCR in LUAD and normal lung epithelial cell lines. The direct interaction between miR-3648 and SOCS2 mRNA was identified through luciferase reporter and RNA pull-down assays. Cell viability, migration, and invasion were examined using cell functional assays. MiR-3648 was found to be overexpressed in LUAD cells and tissues. Overexpression of miR-3648 significantly enhanced cell proliferation, migration, and invasion abilities in LUAD cells. Furthermore, SOCS2 was targeted by miR-3648, and co-transfection of a miR-3648 inhibitor or si-SOCS2 reversed the suppressive effects of SOCS2 in PC9 and A549 cells. miR-3648 enhanced the proliferation and promoted migration and invasion of LUAD by inhibiting SOCS2. In conclusion, our results indicate that miR-3648 plays a pivotal role in LUADe progression and might thus provide a novel therapeutic strategy for patients with LUAD.

## Introduction

Lung cancer ranks first in both incidence (11.6% of the total cancer cases) and cancer-related deaths (18.4% of the total cancer-related deaths) for both sexes combined [1]. In China, lung cancer is the most frequent and fatal cancer, with an incidence rate of 17.1% and a mortality rate of 21.7% in 2015 [2]. In addition, lung cancer morbidity among nonsmokers in China is notably higher than that in other countries, especially in women [3]. Lung adenocarcinoma (LUAD) is the most common histologic subtype of lung cancer and accounts for approximately 40% of lung cancers [4]. Currently, surgery is the most common therapy for patients with early stage LUAD, with or without perioperative chemotherapy (such as erlotinib, an epidermal growth factor receptor tyrosine kinase inhibitor, EGFR TKI), immunotherapy (such as bevacizumab), or radiotherapy (such as stereotactic body radiation) [5–8]. In addition to platinum-based dual therapy with or without bevacizumab, patients with advanced LUAD also benefit from molecular targeted therapies such as gefitinib, erlotinib, afatinib, osimertinib (EGFR TKIs), and crizotinib, ceritinib, and alectinib (anaplastic lymphoma kinase inhibitor, ALK inhibitors), among others [9,10]. Despite extensive research, tumor resistance remains a fundamental challenge in improving patient outcomes [11]. Therefore, there is an urgent need to identify more treatments that are effective for LUAD.

MicroRNAs (miRNAs, miRs) have been associated with cancer progression through gene silencing [12]. The influence of miR-3648 on cancer has only been studied in the last three years. Several studies have found that miR-3648 exerts an essential influence on the survival, migration, and invasion of cancer cells [13,14]. miR-3648 has been demonstrated to be a key tumor promoter in cervical [15], prostate [13], bladder [14,16] and breast cancers [17]. However, the influence of miR-3648 on lung cancer has not yet been reported. Therefore, this study aimed to analyze the effect of miR-3648 on LUAD.

In this study, bioinformatics analysis revealed that suppressor of cytokine signaling 2 (SOCS2) may be a target of miR-3648. SOCS2 is located on chromosome 12q22 and is composed of 12 exons. SOCS2 encodes a member of the SOCS family. SOCS family members function as cytokine-inducible negative regulators of cytokine receptor signaling through Janus kinase/signal transducer (JAK/STAT) and activation of the JAK/STAT pathway [18]. SOCS2 inhibition was found to promote progression and metastasis in colorectal [19], prostate [20], and breast cancers [21], whereas it inhibits the progression and metastasis of ovarian [22] and gastric cancer [23]. Furthermore, four studies have investigated the tumor-suppressing role of SOCS2 in LUAD [24–27]. However, the interaction between miR-3648 and SOCS2 in LUAD cells has not been studied.

Based on bioinformatics analysis and previous studies, we hypothesized a novel interaction between miR-3648 and SOCS2 and aimed to study the effects of miR-3648 and SOCS2 on LUAD. Our study might contribute to a better understanding of LUAD involving both miR-3648 and SOCS2.

## Materials and methods

### Bioinformatics analysis

Two miRNA microarrays (GSE135918 and GSE68951) containing miRNA expression data from LUAD and normal samples from GEO datasets (https://www.ncbi.nlm.nih.gov/geo/) were used to identify the differentially expressed miRNAs (DE-miRNAs) with adjusted P < 0.05, |logFC | ≥ 1.5 and adjusted P < 0.05, |logFC| ≥ 1, respectively. TargetScan (http://www.targetscan.org/vert_71/) was used to identify the target genes of miR-3648. GSE85841 from GEO datasets was used to identify the differentially expressed genes (DEGs) with adjusted P < 0.01, logFC < −1.5. The expression of screened genes in LUAD samples was analyzed using GEPIA (http://gepia.cancer-pku.cn/index.html).

### Clinical samples

LUAD and paired noncancerous tissue samples were obtained from 38 patients at our hospital. Clinical tissue samples were handled in strict accordance with the Helsinki Declaration, and the ethics committee of our hospital (WHSHIRB-K-2019011) approved the project. All volunteers provided written informed consent, and their clinical characteristics are listed in Table 1.

**Table 1.** Clinical characteristics of the lung cancer patients in this study (N = 38)

Parameter	Value[No.(%)]
Age(years)	
≥60	20(52.6)
<60	18(47.4)
Sex	
Male	22(57.9)
Female	16(42.1)
Tumor size	
≥5 cm	17(44.7)
<5 cm	21(55.3)
Smoking history	
Never smokers	25(65.8)
Current or former	13(34.2)
Stage	
I	6(15.8)
II	2(5.3)
III	14(36.8)
IV	16(42.1)
Location	
Central	26(68.4)
Peripheral	12(31.6)

### Cell culture and transfection

Human LUAD cell lines PC9, A549, Calu-3, and DV-90, and the normal lung epithelial cell line BeAS-2B were obtained from the American Type Culture Collection. They were cultured in RPMI 1640 (Sigma, USA), F12K (Sigma), minimum essential medium (MEM; Sigma), or bronchial epithelial growth medium (BEGM; Clonetics, USA) containing 10% fetal bovine serum (Gibco Laboratory, USA), and 1% penicillin/streptomycin (Sigma) in a humidified incubator at 37°C with 5% CO_2_.

miR-3648 mimics, miR-3648 inhibitor, mimics negative control (NC), NC inhibitor, si-SOCS2, and si-NC were acquired from Gene Copoeia (Guangzhou, China). PC9 and A549 cells were inoculated in 6-well plates at a seeding density of 3 × 10^4^ cells/mL and transfected with the above molecules for 48 h. Transfection was performed using Lipofectamine™ 2000 reagent (11,668,027, Thermo Fisher, USA) according to the manufacturer’s instructions.

### Real-time quantitative PCR (RT-qPCR)

Total miRNA content was extracted with miRNeasy Tissue/Cells Advanced Mini Kit (217,604, Qiagen, Germany), following the manufacturer’s instructions, and the miRNA purity was determined using RNA gel electrophoresis. Reverse transcription of miR-3648 was performed using miRNA First Strand cDNA Synthesis (B532451, Sangon Biotech, China) followed by quantitative PCR using TB Green® Premix Ex Taq™ II (RR820A, Takara, Japan). The 2^−ΔΔCt^ method and U6 as the reference gene was used to calculate the RNA content [28]. Sangon Biotech (Shanghai, China) supplied all related primers, and the primer design is shown in Table 2.

**Table 2.** PCR primers used in this study

Gene	Forward primer	Reverse primer
GAPDH	5ʹ-TGCACCACCAACTGCTTAGC-3’	5ʹ-GGCATGGACTGTGGTCATGAG-3’
SOCS2	5ʹ-TCGGTCAGACAGGATGGTACT-3’	5ʹ-AGTTCCTTCTGGTGCCTCTTT-3’
miR-3648	5ʹ-CACGCAGCCGCGGGGAT-3’	5ʹ-CCAGTGCAGGGTCCGAGGTA-3’
U6	5ʹ-GCTTCGAGGCAGGTTACATG −3ʹ	5ʹ-GCAACACACAACATCTCCCA-3’

### Cell viability assay

The Cell Counting Kit-8 (CCK-8) method was used to assess cell viability, as described previously [29]. Briefly, transfected PC9 and A549 cells were inoculated into 96-well plates at a density of 4,000 cells/well. The cells were cultured for 12, 24, 48, and 72 h, and then 10 μL of CCK-8 solution (KGA317, Keygen Biotech, China) was added to the cells. Following incubation for 4 h at 37°C, the absorbance of each well was measured at a wavelength of 450 nm using a microplate reader and the time-growth curve was plotted.

### 5-bromodeoxyuridine (BrdU) uptake assay

To detect cell proliferation, we performed a BrdU DNA incorporation assay as reported previously [30]. Briefly, 4 × 10^4^/mL PC9 and A549 cells were inoculated in a 96-well plate and incubated overnight. BrdU solution (10 μL/well; ab126556, Abcam, UK) was added and the cells were incubated for 1 h. Subsequently, fixing solution was added to the wells to denature the cellular DNA. After discarding the fixing solution and washing the cells, 100 μL anti-BrdU primary antibody solution was added to each well and further incubated for 1 h. After the primary antibody solution was discarded and washed, 100 μL of goat anti-mouse IgG secondary antibody (peroxidase conjugated) was added to each well and incubated for 30 min. Next, 100 µL of TMB solution (peroxidase substrate) was added to each well and the cells were incubated in the dark for 0.5 h. Proliferative cells that incorporate BrdU appear blue, and the color intensity represents the proliferative ability. For further quantitative analysis of cell proliferation, 100 µL of termination reaction reagent was added, and the absorbance was determined at 450 nm using a microplate reader (BioTek Instruments, USA). All procedures were performed at room temperature and each experiment was repeated three times.

### Wound healing assay

PC9 and A549 cells (2 × 10^5^ cells/well) were inoculated into 6-well plates and incubated at 37°C for 24 h until confluent. Then, the cell monolayers were scraped along the center of each well using a sterile 200 μL tip, and the plates were rinsed with phosphate buffered saline (PBS) to eliminate detached cells. The transfected cells were incubated for 24 h. Images of each group at the scratch sites were acquired at 0 and 24 h, respectively, under an inverted microscope at a magnification of 10×, and cell migration was quantified using ImageJ software (National Institute of health, USA) as reported previously [31].

### Transwell invasion assay

Transwell chambers (8.0 μm pore membranes; 3422, Corning, USA) were utilized to detect cell invasion ability as described previously [31]. Briefly, Matrigel was added to the upper chamber and allowed to solidify for 6 h at 37°C. Then, 1 × 10^5^ resuspended transfected cells were added to the upper chamber with 200 μL serum-free medium, and 800 μL medium supplemented with 10% FBS was added to the lower chamber. After incubation for 24 h at 37°C, the noninvasive cancer cells that remained on the upper surface of the membrane were removed with wet cotton swabs. The invaded cells on the bottom of the membrane were fixed in 4% paraformaldehyde for 0.5 h and stained with 0.5% crystal violet solution for 0.5 h at room temperature. Then the cells were rinsed with PBS and the stained cells were photographed and quantified using a light microscope (Olympus, Japan).

### Luciferase reporter gene assay

The recombinant vectors containing wild-type or mutant SOCS2 mRNA binding sites, were constructed and supplied by Gene Copoeia (Guangzhou, China) as described previously [32]. First, we transfected the plasmids into PC9 and A549 cells using the Lipofectamine™ 2000 reagent. Then, we transfected miR-3648 mimic or NC into the transfected cells using Lipofectamine™ 2000 reagent according to the manufacturer’s instructions. After culturing for 48 h, the cells were collected and lysed with lysis buffer. Then, to quantitatively evaluate the inhibitory effect of the miRNA on target genes, we determined the relative luciferase activity, which represents the mRNA content, using the Dual-Luciferase® Reporter Assay System (E1910, Promega, USA) following the manufacturer’s instructions.

### RNA pull-down assay

PC9 and A549 cells (5 × 10^6^) were incubated on ice with 500 μL solution containing 0.05% NP-40, 25 mM Tris-HCl, 2.5 mM EDTA, 70 mM KCl, 1× protease inhibitor cocktail, and 80 U/mL RNase inhibitor for 20 min. After centrifugation, the cellular extracts were mixed with biotinylated miR-3648 (bio-miR-3648) or biotinylated NC (bio-NC) for 2 h. Then, the cell lysates were conjugated with streptavidin magnetic beads, followed by incubation at 4°C for 3 h. Next, the bound RNAs in the pull-down complex were isolated by washing the beads with reaction buffer and the pull-down RNA expression were determined by RT-qPCR as previously described [33].

### Western blotting

Western blot assay was performed as previously described [34]. Cellular proteins were extracted with radioimmunoprecipitation assay (RIPA) lysis buffer, and the total protein in various groups were quantified using a spectrophotometer to determine equal concentration. Proteins (20 μg) were loaded onto a 10% sodium dodecyl sulfate polyacrylamide gel electrophoresis (SDS-PAGE) gel and transferred to polyvinylidene difluoride (PVDF) membranes. After blocking in 5% nonfat milk for 0.5 h, the membranes were incubated with primary antibodies against SOCS2 (1:1000, 2779 T, Cell Signaling Technology, USA) and GAPDH (1:1000, 5174 T, Cell Signaling Technology) overnight at 4°C. Then, the membranes were washed with 1× TBST three times, followed by a 1 h incubation with goat anti-rabbit IgG, light-chain-specific antibody (HRP conjugate; 1:4000, 98,164 T, Cell Signaling Technology). We used SignalFire Plus ECL Reagent (12630S, Cell Signaling Technology) to enhance the signals from the protein bands and quantified the intensities of the bands using a Bio-Rad ChemiDoc MP imager (Bio-Rad, USA).

### Statistical analyses

The data were analyzed using SPSS (SPSS, USA) and GraphPad Prism v7.0 (GraphPad Software, USA). Data were obtained from at least three independent experiments and are presented as the mean ± standard deviation (SD). We identified significance of differences using the Student’s *t*-test method. p < 0.05 was considered statistically significant.

## Results

In this study we tested the hypothesis that miR-3648 interacts with SOCS2 in LUAD cells. We first dissected the positive role of miR-3648 in LUAD *in vitro*. The interaction of miR-3648 and SOCS2 in LUAD progression was also verified. Our findings may enrich our understanding of LUAD progression.

### Identification of miR-3648 and SOCS2 in LUAD

**Figure 1.**
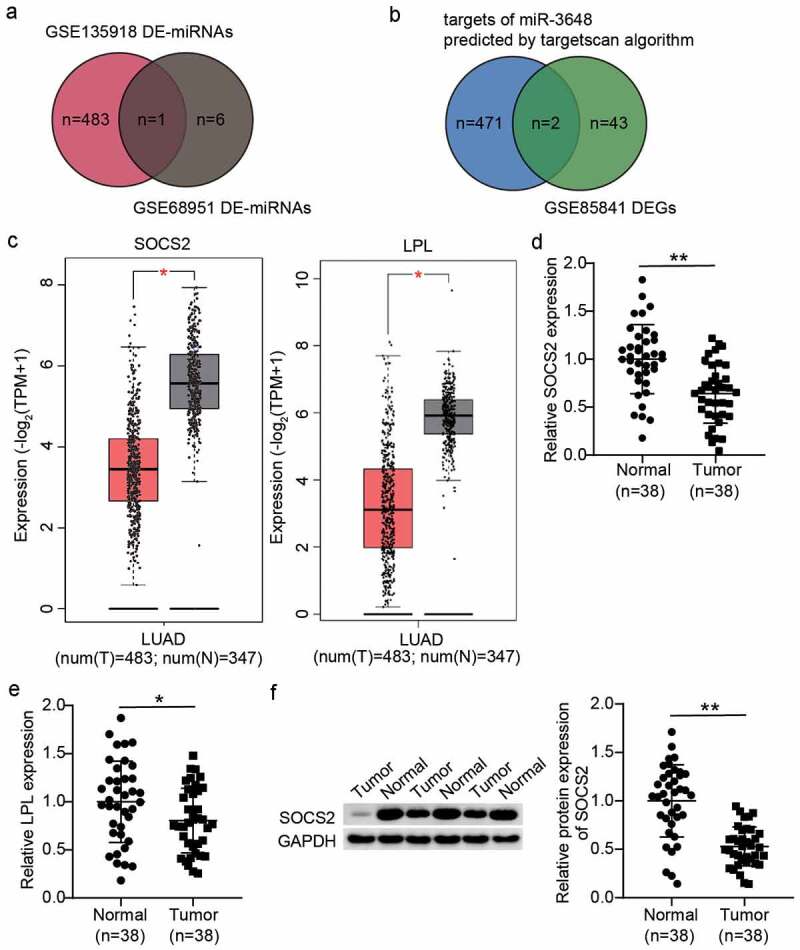
**The identification of miR-3648 and SOCS2 in LUAD**. A. The identification of miR-3648 by intersecting the differentially expressed miRNA (DE-miRNA) lists of GSE135918 (adjusted P < 0.05, |logFC|≥1.5) and GSE68951 (adjusted P < 0.05, |logFC|≥1) data series. B. The identification of LPL and SOCS2 in LUAD by intersecting the gene lists of Targetscan-predicted targets of miR-3648 and the differentially expressed genes (DEGs) of GSE85841. C. The expression box plot of SOCS2 and LPL in LUAD via GEPIA data analysis. Num: number; T: tumor; N: normal controls. D-E. Verification of SOCS2 expression (d) and LPL expression (e) in LUAD tissues and matched normal tissues. F. Western blotting analysis of SOCS2 expression in LUAD tissues and matched normal tissues. (d-f) *P < 0.05, **P < 0.001 vs. normal tissues.

By analyzing the GSE135918 and GSE68951 miRNA data series, we identified 484 DE-miRNAs in the former and seven in the latter data series. A common miRNA identified in both the datasets was miR-3648 (Figure 1(a)). A previous study reported that miR-3648 is upregulated and promotes bladder cancer mobility phenotypes [14]. miR-3648 has also been reported to enhance growth of HeLa cells [15]. However, its role in lung cancer has not yet been studied. To identify the potential downstream target gene of miR-3648, we intersected the predicted targets of miR-3648 using the TargetScan algorithm and the DEGs in LUAD by analyzing GSE85841. Lipoprotein lipase (LPL) and SOCS2 were identified as the overlapping genes in the two datasets (Figure 1(b)). The GEPIA database also demonstrated that SOCS2 and LPL were significantly downregulated in LUAD (Figure 1(c)). Quantification of SOCS2 and LPL expression in LUAD and normal tissues showed that SOCS2 expression showed more difference between tumor and normal tissues compared to that of LPL (Figure 1(d-e)). Therefore, we chose SOCS2 for the subsequent assays. Previously, SOCS2 was reported to inhibit proliferation and promote the apoptotic phenotype in lung cancer [25,26,35]. Because mobility is a significant feature of cancer cells, studies on the effects of SOCS2 on cancer cell mobility phenotypes are important. Therefore, we hypothesized that miR-3648 may regulate lung cancer cell phenotypes by targeting SOCS2.

### miR-3648 promotes proliferation of LUAD cells

**Figure 2.**
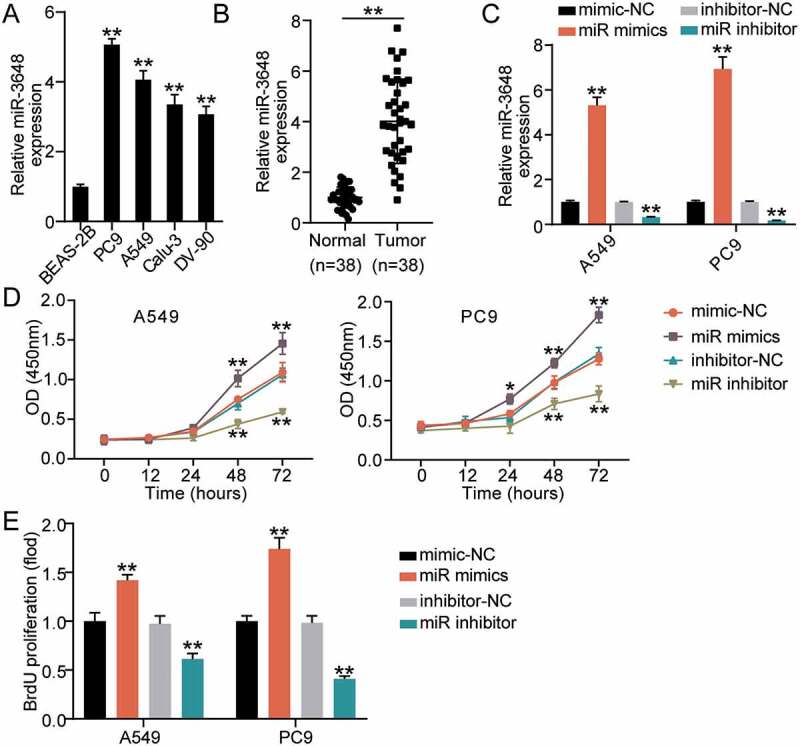
**miR-3648 promoted proliferation in LUAD cells** A. The expression of miR-3648 in four LUAD cell lines (PC9, A549, Calu-3 and DV-90) and normal lung epithelial cell line (BeAS-2B) was measured by RT-qPCR. B. Expression of miR-3648 in LUAD and adjacent normal tissues. N = 38 C. The expression of miR-3648 in PC9 and A549 cells transfected with miR mimics, miR inhibitor, mimic-NC and inhibitor-NC was measured by RT-qPCR. D. Cell viability was observed in PC9 and A549 cell lines after transfection with miR mimics, miR inhibitor, mimic-NC and inhibitor-NC at 0, 12, 24, 48, and 72 hours via CCK-8 assay. E. Cell proliferation was observed in PC9 and A549 cell lines after transfection with miR mimics, miR inhibitor, mimics-NC and NC inhibitor via BrdU incorporation assay. *P < 0.05, **P < 0.001 compared with BeAS-2B (b) or mimic-NC(c-e) group, Student’s t-test.

To evaluate the effect of miR-3648 in LUAD, we first analyzed the expression of miR-3648 in normal lung epithelial cells and LUAD cell lines using RT-qPCR. The results showed that, compared to normal lung epithelial cells (BeAS-2B), the expression of miR-3648 was significantly higher in LUAD cell lines. PC9 and A549 cell lines showed approximately 4- and 3-fold increase, respectively, in miR-3648 expression (Figure 2(a)). Therefore, we selected PC9 and A549 cell lines for follow-up studies. Similar to LUAD cell lines, the expression of miR-3648 in LUAD tissues from 38 patients who were diagnosed with LUAD was upregulated 3-fold compared to paired noncancerous tissues (Figure 2(b)). Changes in miR-3648 expression in PC9 and A549 cells were analyzed following transfection with miR-3648 mimics and inhibitors. The results demonstrated that miR-3648 level was significantly upregulated in A549 and PC9 cells following transfection with miR-3648 mimics, and increased by approximately 4- and 6- fold, respectively. Similarly, miR-3648 expression was significantly downregulated following transfection with the miR-3648 inhibitor (Figure 2(c)). CCK8 experiments showed that cell proliferation and viability were enhanced after transfection with miR-3648 mimics (Figure 2(d)). The BrdU experiment also demonstrated similar result (Figure 2(e)).

### miR-3648 promotes migration and invasion of LUAD cells

**Figure 3.**
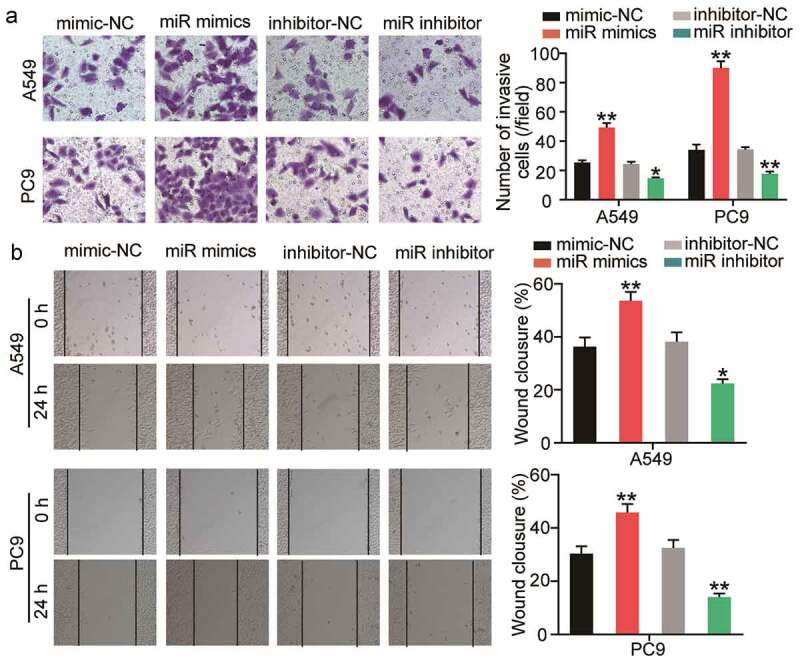
**miR-3648 promoted cell migration and invasion in LUAD cells** A. Cell invasion was evaluated in A549 and PC9 cell lines after transfection with miR mimics, miR inhibitor, mimic-NC and inhibitor-NC via Transwell assay. B. Cell migration was examined in A549 and PC9 cell lines after transfection with miR mimics, miR inhibitor, mimic-NC and inhibitor-NC via wound healing assay. *P < 0.05, **P < 0.001 compared with mimic-NC group, Student’s t-test.

To evaluate the changes in invasive and migratory abilities of the cells, we performed Transwell invasion and wound healing assays. The results of the Transwell invasion assay demonstrated that transfection with miR-3648 mimics significantly promoted cell invasion by approximately 1- and 2-fold in A549 and PC9 cells, respectively (Figure 3(a)). After incubation for 24 h, the scratches in the miR-3648 mimic groups were significantly reduced, and quantitative analysis results showed that the cell migration ability was significantly enhanced by approximately 0.4- and 0.5-fold in A549 and PC9 cells, respectively (Figure 3(b)).

### SOCS2 is a downstream target gene of miR-3648

**Figure 4.**
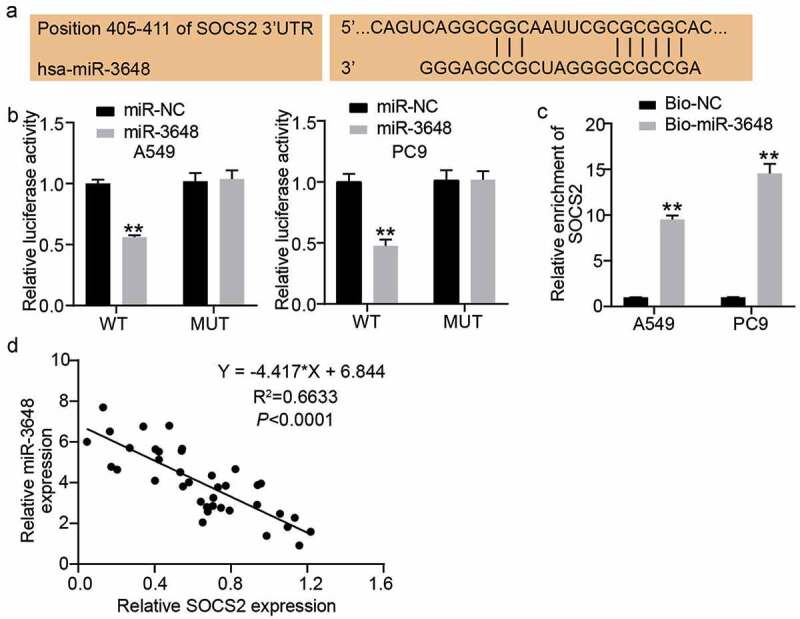
**SOCS2 was a downstream target gene of miR-3648** A. The binding site of SOCS2 mRNA 3ʹUTR for miR-3648. B. The target relationship between miR-3648 and SOCS2 was measured by luciferase reporter assay. C. The target relationship between miR-3648 and SOCS2 was measured by RNA pull-down assay. D. Correlation between miR-3648 and SOCS2. *P < 0.05, **P < 0.001 compared with miR-NC (b) or Bio-NC (c) group, Student’s t-test.

Based on the above-mentioned prediction from bioinformatics analysis, we next sought to investigate the interaction between SOCS2 and miR-3648. We first obtained the complementary sequences of the 3ʹ UTR of SOCS2 mRNA and miR-3648 by interrogating TargetScan Human 7.2 (Figure 4(a)). Subsequently, we performed luciferase reporter and RNA pull-down assays to examine the direct interaction between SOCS2 mRNA and miR-3648. The fluorescence intensity was significantly decreased by approximately 0.5-fold in the SOCS2 wild-type sequence in both A549 and PC9 cells transfected with miR-3648 mimics (Figure 4(b)). RNA pull-down experiments further confirmed that miR-3648 binds to SOCS2 mRNA, with 9- and 13-fold increase in enrichment of SOCS2 in A549 and PC9 cells, respectively (Figure 4(c)). We also found that miR-3648 expression was negatively correlated with SOCS2 expression (Figure 4(d).

### miR-3648 reverses the suppressive effect of SOCS2 on the malignant phenotype of LUAD cells

**Figure 5.**
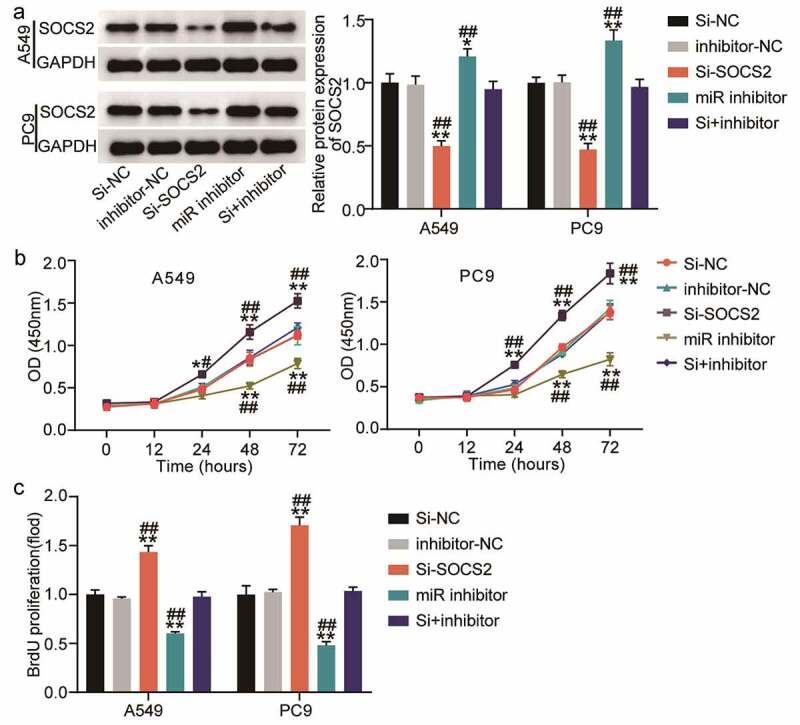
**miR-3648 reverse the suppression effects of SOCS2 on LUAD cell malignancy phenotypes** A. The expression of SOCS2 protein was detected by Western blot in A549 and PC9 cells after transfection with miR-3648 inhibitor, si-SOCS2, si-NC, inhibitor-NC and co-transfection with miR-3648 inhibitor and si-SOCS2. B. Cell viability was measured by CCK-8 assay in A549 and PC9 cells after transfection with miR-3648 inhibitor, si-SOCS2, si-NC, NC inhibitor and co-transfection with miR-3648 inhibitor and si-SOCS2. C. Cell proliferation was measured by BrdU incorporation assay in PC9 and A549 cells after transfection with miR-3648 inhibitor, si-SOCS2, si-NC, NC inhibitor and co-transfection with miR-3648 inhibitor and si-SOCS2. *P < 0.05, **P < 0.001 compared with si-NC group; ^#^P < 0.05, ^##^P < 0.001 compared with si+inhibitor group, Student’s t-test.

To further verify the role of SOCS2 in miR-3648-mediated phenotypic alteration in LUAD cells, we transfected PC9 and A549 cells with miR-3648 inhibitor and si-SOCS2 to identify whether the effect of SOCS2 on LUAD cells was regulated by miR-3648. Western blot assays confirmed that si-SOCS2 and miR-3648 inhibitors were successfully transfected into the cancer cells. Specifically, miR-3648 inhibitor significantly increased the expression of SOCS2, whereas si-SOCS2 significantly decreased the expression of SOCS2 (Figure 5(a). We then performed CCK-8 and BrdU incorporation experiments to evaluate the viability and proliferative ability of the cells. The results showed that si-SOCS2 attenuated the viability and proliferative ability of PC9 and A549 cells, whereas co-transfection with miR-3648 inhibitor impaired the effect of SOCS2 on viability and proliferative ability of PC9 and A549 cells (Figure 5(b-c)).

### miR-3648 reverses the suppressive effects of SOCS2 on LUAD cell metastasis

**Figure 6.**
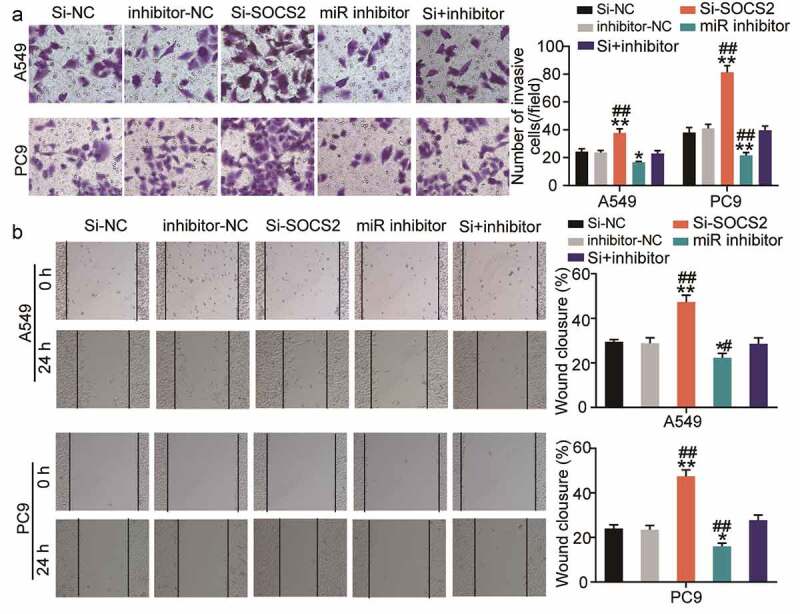
**miR-3648 reverse the suppression effects of SOCS2 on LUAD cell metastasis** A. Cell invasion was evaluated via transwell assay in A549 and PC9 cells after transfection with miR-3648 inhibitor, si-SOCS2, si-NC, inhibitor-NC and co-transfection with miR-3648 inhibitor and si-SOCS2. B. Cell migration was examined in PC9 and A549 cell lines after transfection with miR-3648 inhibitor, si-SOCS2, si-NC, inhibitor-NC and co-transfection with miR-3648 inhibitor and si-SOCS2 via wound healing assay. *P < 0.05, **P < 0.001 compared with si-NC group; ^#^P < 0.05, ^##^P < 0.001 compared with si+inhibitor group, Student’s t-test.

In addition to proliferation, we also investigated the effect of SOCS2 on the miR-3648-mediated invasive and migratory abilities of the cancer cells. We found that si-SOCS2 significantly enhanced cell migration and invasion, whereas co-transfection with miR-3648 inhibitor significantly weakened the effect of SOCS2 on PC9 and A549 cells (Figure 6(a-b)).

## Discussion

In the present study, we found that miR-3648 was overexpressed in LUAD cells and tissues. Functional assays showed that miR-3648 overexpression significantly enhanced cell proliferation, and promoted migration and invasion abilities of LUAD cells. Using luciferase reporter assay, we also found that SOCS2 was the target of miR-3648. Furthermore, co-transfection of miR-3648 inhibitor and si-SOCS2 reversed the suppressive effects of SOCS2 on the malignant phenotype of LUAD cells. These results revealed the potential role of miR-3648 in promoting LUAD progression by downregulating SOCS2 expression.

Although several studies have been conducted to identify the role of miR-3648 in tumor progression, research on LUAD is limited. Rashid et al. found that increased expression of miR-3648 induced by endoplasmic reticulum (ER) stress downregulated adenomatous polyposis coli 2 (APC2) and promoted the proliferation of cervical cancer and other cells [15]. miR-3648 facilitates prostate cancer cell proliferation by inhibiting APC2, which leads to the activation of the Wnt/β-catenin pathway by increasing cyclin D1 and cyclin E1 expression and decreasing p21 expression [13]. miR-3648 is overexpressed and promotes invasion and metastasis of human bladder cancer by directing transcription factor 21 (TCF21)/kisspeptin 1 (KISS1) axis [14]. In addition, higher expression of miR-3648 was correlated with shorter overall survival time in patients with bladder cancer [16]. miR-3648 was recently identified to positively correlate with the recurrence score (RS) of breast cancer, likely owing of its contribution to cancer growth, aggressiveness, and response to therapies [17]. Therefore, miR-3648 serves as a promoter in various cancers.

In the current study, we observed that miR-3648 was notably upregulated in LUAD, suggesting that miR-3648 may act as an oncomiR in LUAD. To determine the effect of miR-3648 on cancer pathogenesis, we performed *in vitro* studies to evaluate the effects of miR-3468 in LUAD, based on previous literature. We found that miR-3468 promoted cell proliferation, inhibited apoptosis, and promoted migration and invasion, consistent with the results of previous studies.

The results of our bioinformatics analysis indicated that SOCS2 was a direct target of miR-3648. SOCS2 acts as a tumor suppressor that regulates cell fate in many cancers [36–38]. SOCS2 expression, which was inhibited by exosomal miR-3613-3p promotes proliferation, metastasis, and drug resistance of breast cancer cells, and SOCS2 is expressed at low levels in breast cancer tissues [39]. Low SOCS2 levels have also been reported in patients with LUAD [40]. Downregulation of SOCS2 by different miRNAs has been found to promote proliferation [24], migration, and metastasis [25], and decrease apoptosis of lung cancer cells [24]. Consistent with previous studies, we observed lower expression of SOCS2 in human tumor tissues than in normal tissues. To confirm our hypothesis that miR-3648 mediates its oncogenic effects by negatively regulating SOCS2, we transfected cancer cells with a miR-3648 inhibitor with or without si-SOCS2 and performed a series of experiments. Our results confirmed that miR-3648 directly binds to the 3ʹ UTR region of SOCS2, and also indicated that miR-3648 inhibitor increases the expression of SOCS2, suggesting that miR-3648 downregulates SOCS2. Furthermore, co-transfection of miR-3648 inhibitor and si-SOCS2 partially reversed the antitumor effects mediated by the miR-3648 inhibitor. Thus, our results showed that miR-3648 might promote cancer by downregulating SOCS2 expression. In contrast, in lung cancer, SOCS2 is involved in a complex regulatory network. For example, SOCS2 is targeted by miR-578, miR-875, and miR-H9-5p and suppresses the malignant behavior of non-small cell lung cancer cells [24,25,41]. Furthermore, SOCS2 suppresses insulin-like growth factor 1 (IGF1)-driven migratory and invasive behavior of LUAD cells. In future studies, we will focus on the SOCS2-driven complex regulatory networks in LUAD.

Although our study is significant and novel, it also has limitations. We only performed relevant experiments at the cellular level and did not perform any animal experiments. Furthermore, the progression of carcinoma is a complex process involving a network of signaling pathways. We have not researched the specific mechanisms or relevant signaling pathways thoroughly, but only focused on the interaction between miR-3648 and SOCS2 in LUAD. Therefore, in future studies we will conduct animal experiments and focus on the study of the signaling pathways involved.

## Conclusion

The present study demonstrated that miR-3648 promotes the proliferation, migration, and invasion of LUAD by downregulating its target SOCS2. Our results indicate that miR-3648 may play a pivotal role in the progression of LUAD and provides a novel therapeutic strategy for patients with LUAD.

## Data Availability

The datasets used and/or analyzed during the current study are available from the corresponding author on reasonable request.

## Highlights

- 1. miR-3648 is overexpressed in LUAD cells and tissues
- 2. miR-3648 overexpression promotes malignant behavior of LUAD cells
- 3. miR-3648 targets SOCS2 to regulate LUAD progression *in vitro*
